# Pancreatic carcinoma, pancreatitis, and healthy controls: metabolite models in a three-class diagnostic dilemma

**DOI:** 10.1007/s11306-012-0476-7

**Published:** 2012-11-06

**Authors:** Alexander Benedikt Leichtle, Uta Ceglarek, Peter Weinert, Christos T. Nakas, Jean-Marc Nuoffer, Julia Kase, Tim Conrad, Helmut Witzigmann, Joachim Thiery, Georg Martin Fiedler

**Affiliations:** 1 Center of Laboratory Medicine, University Institute of Clinical Chemistry, Inselspital—Bern University Hospital, Inselspital INO F 502/UKC, 3010 Bern, Switzerland; 2 Institute of Laboratory Medicine, Clinical Chemistry, and Molecular Diagnostics, University Hospital Leipzig, 04103 Leipzig, Germany; 3 Leibniz Supercomputing Centre, Bavarian Academy of Sciences and Humanities, Boltzmannstr. 1, 85748 Garching, Germany; 4 Laboratory of Biometry, University of Thessaly, Fytokou Str., N. Ionia, 38446 Magnesia, Greece; 5 Department of Hematology, Oncology and Tumor Immunology, Campus Virchow Clinic, and Molekulares Krebsforschungszentrum, Charité—Universitätsmedizin, Augustenburger Platz 1, 13353 Berlin, Germany; 6 Department of Mathematics, Free University of Berlin, Arnimallee 6, 14195 Berlin, Germany; 7 Clinic of Visceral Surgery, University Hospital Leipzig, Liebigstr. 20, 04103 Leipzig, Germany; 8 Center of Laboratory Medicine, University Institute of Clinical Chemistry, Inselspital—Bern University Hospital, Inselspital INO F 610/UKC, 3010 Bern, Switzerland; 9 Center of Laboratory Medicine, University Institute of Clinical Chemistry, Inselspital—Bern University Hospital, Inselspital INO F 603/UKC, 3010 Bern, Switzerland

**Keywords:** Pancreatic cancer, Metabolomics, Amino acids, Modeling, Marker panels

## Abstract

Metabolomics as one of the most rapidly growing technologies in the “-omics” field denotes the comprehensive analysis of low molecular-weight compounds and their pathways. Cancer-specific alterations of the metabolome can be detected by high-throughput mass-spectrometric metabolite profiling and serve as a considerable source of new markers for the early differentiation of malignant diseases as well as their distinction from benign states. However, a comprehensive framework for the statistical evaluation of marker panels in a multi-class setting has not yet been established. We collected serum samples of 40 pancreatic carcinoma patients, 40 controls, and 23 pancreatitis patients according to standard protocols and generated amino acid profiles by routine mass-spectrometry. In an intrinsic three-class bioinformatic approach we compared these profiles, evaluated their selectivity and computed multi-marker panels combined with the conventional tumor marker CA 19-9. Additionally, we tested for non-inferiority and superiority to determine the diagnostic surplus value of our multi-metabolite marker panels. Compared to CA 19-9 alone, the combined amino acid-based metabolite panel had a superior selectivity for the discrimination of healthy controls, pancreatitis, and pancreatic carcinoma patients $[ {\text{volume under ROC surface}}\;\left( {\text{VUS}} \right) = 0. 8 9 1 { }\left( { 9 5\,\% {\text{ CI }}0. 7 9 4- 0. 9 6 8} \right)].$ We combined highly standardized samples, a three-class study design, a high-throughput mass-spectrometric technique, and a comprehensive bioinformatic framework to identify metabolite panels selective for all three groups in a single approach. Our results suggest that metabolomic profiling necessitates appropriate evaluation strategies and—despite all its current limitations—can deliver marker panels with high selectivity even in multi-class settings.

## Introduction

Pancreatic cancer is the fourth leading cause of cancer death in the United States, and most patients diagnosed with pancreatic cancer develop clinical symptoms usually late in the course of the disease (Lowenfels and Maisonneuve 2006). Therefore, only 20 % of patients can be treated with a potentially curative therapy and only about 3–5 % survive at least 5 years (Michl et al. 2006). For these patients, time, especially the so called ‘biomarker lead time’ between the onset of asymptomatic cancer still localized to the organ of origin and clinical diagnosis (Konforte and Diamandis 2012), is crucially important (Hazelton and Luebeck 2011). Although recent modeling studies have illustrated that blood-based biomarkers might provide a successful tool for the early detection and differentiation of premalignant lesions, substantial methodological enhancements of unanticipated extent (Burgess 2012) are still required. Yachida et al. (2010) demonstrated a latency of about 17 years from the initiating mutation to pancreatic cancer death. Similarly, Hori and Gambhir (2011) stated “that shedding rates of current clinical blood biomarkers are likely 10^4^-fold too low to enable detection of a developing tumor within the first decade of tumor growth” and suggested to increase sensitivity and specificity by introducing multi-marker panels of up to 10 biomarkers. In a proof-of-principle study for evaluating the utility of multiplexed circulating biomarkers, Brand et al. (2011) investigated the selectivity of 83 proteins and their combinations. Two panels consisting of CA 19-9, ICAM-1, and OPG, as well as CA 19-9, CEA, and TIMP-1 were found to discriminate pancreatic cancer patients from healthy control subjects and from patients with benign pancreatic conditions, respectively. Since the cohorts in this study were compared separately, an integral model encompassing all three disease states was not obtained. Whereas Brand et al. (2011) focused on known tumor markers, tumor-associated peptides, etc., other studies have employed several of the emerging “-omics” subspecialties, such as proteomics (Fiedler et al. 2009), transcriptomics (Zhang et al. 2010), and—as the probably closest to the “bedside” (Van and Veenstra 2009)—metabolomics (Bathe et al. 2011; Ceglarek et al. 2009; Nishiumi et al. 2010; OuYang et al. 2011; Urayama et al. 2010; Zhang et al. 2011). The latter bears the chance to learn from the intricacies that have plagued “-omics” researchers over the last years, standardization (Van and Veenstra 2009), data processing (Blekherman et al. 2011) and data interpretation (Kholodenko et al. 2012), amongst others.

In this study, we addressed these challenges by using a three-class study design. We collected highly standardized samples of pancreatic cancer patients, subjects with pancreatitis, and healthy controls. Following tandem mass spectrometric metabolite profiling, we evaluated the differences between groups and applied Bayesian methodology to identify multi-metabolite models as “meta-markers”, which are selective for each of the three study groups and provide improved diagnostic performance compared to CA 19-9, the conventional tumor marker.

## Materials and methods

### Patients and samples

We recruited patients suffering from pancreatic cancer ($n = 40$), healthy controls ($n = 40$), and patients hospitalized due to acute pancreatitis ($n = 2 6$) at the University Hospital of Leipzig in the context of previously published studies (Fiedler et al. 2009; Leichtle et al. 2012). We collected cubital vein fasting samples of cancer patients and healthy controls in two independent sets. Additionally, we collected fasting serum samples of 26 patients with pancreatitis as inflammatory control group (A, C, and D, $n_{\text{total}} = 10 6$; Table 1). We adjusted subjects according to age and gender and performed blood sampling from patients before the initiation of specific therapy. Diagnosis of pancreatic cancer was confirmed by histologic examination in all cases. Healthy controls showed no evidence of actual disease in physical examination and routine laboratory testing [alkaline phosphatase, bilirubin, C-reactive protein, CA 19-9, CEA, creatinine, γ-glutamyltransferase, transaminases (Roche Modular, Germany)]. Pancreatitis patients were diagnosed clinically without proof of pancreatic carcinoma during the study period. Serum samples were collected and stored (at −80 °C) using standardized techniques and protocols (Baumann et al. 2005).

**Table 1:** Baseline data for the two study sets (A and C) as well as the pancreatitis cohort (D)

Unit or stage	Total cohort						
	Set A		Set C		Set D	Sets A and C	
	Tumor	Control	Tumor	Control	Pancreatitis	Tumor	Control
*N*	20	20	20	20	26	40	40
Gender (m/f)	10/10	10/10	10/10	10/10	22/4^#^	20/20	20/20
Stage							
I	1	–	2	–	–	3	–
II	6	–	5	–	–	11	–
III	1	–	0	–	–	1	–
IV	12	–	11	–	–	23	–
N.c.	0	–	2	–	–	2	–
Age (years)	59.0 (47.9–70.5)^a,b^	51.0 (38.0–66.7)^a,c^	*66.0 (43.9*–*71.5)* ^*c,d,e*^	53.5 (33.4–69.5)^d^	43.5 (26.8–66.0)^b,e^	62.0 (45.7–71.0)	52.0 (34.9–70.0)
CA 19-9 (U/mL)^†^	*149.2 (18.9*–*837.9)* ^*f,g,h*^	6.4 (0.6–12.7)^f,i^	*129.9 (0.65*–*701.9)* ^*i,j*^	*7.2 (0.6*–*18.9)* ^*g,j*^	*8.7 (3.1*–*109.1)* ^*h*^	*141.8 (0.7*–*770.8)*	*6.6 (0.6*–*17.1)*
BMI (kg/m^2^)	23.7 (18.8–30.8)	23.3 (17.8–28.4)	24.4 (19.1–28.3)^k^	27.8 (20.6–36.3)^k,l^	22.3 (17.8–27.4)^l^	24.2 (18.3–30.4)	26.5 (19.1–34.9)
Bilirubine (μmol/L)^†^	*11.5 (3.9*–*65.7)*	*9.3 (5.7*–*22.8)*	*15.9 (4.2*–*118.0)*	13.1 (6.0–21.6)	*7.2 (4.3*–*75.5)*	*11.6 (4.0*–*107.0)*	*10.3 (5.8*–*24.1)*
HbA1c (%)^†^	*6.0 (5.0*–*7.8)* ^*m*^	5.2 (4.7–6.1)^m,n,o^	*5.8 (4.4*–*8.9)*	*5.8 (5.1*–*7.8)* ^*n*^	*6.0 (5.1*–*8.6* ^*o*^	*5.9 (4.7*–*9.0)*	*5.5 (4.8*–*7.3)*

Data are shown as numbers or median (percentiles 2.5–97.5 %). Normality was tested with the Anderson–Darling test, non-normally distributed data are denoted in *italics*. Gender distribution in the subgroups was evaluated with a binomial test ($^{\# } \,P = 0.000 5$)

After testing the quantitative variables for homogeneity of variances (Fligner–Killeen test, ^†^ *P* < 0.05), paired differences in the quantitative variables were investigated by Games–Howell testing (variables sharing a common *superscript letter* are significantly different at *P* < 0.05)

*N.c.* not classified

### Chemicals, standards and consumables

Methanol and isopropanol (gradient grade) were purchased from Merck (Darmstadt, Germany). The amino acid isotope labelled standard kit (NSK-A, Cambridge Isotope Laboratories, Andover, USA) was used as internal standard. Water (HPLC grade) was obtained from J. T. Baker (Deventer, Netherlands). The derivatization reagent 3*n* butanolic HCl was made in-house by mixing 4:1 v/v of 1-butanol (for spectroscopy) from Merck (Darmstadt, Germany) and acetyl chloride (p.a.) from Sigma-Aldrich (Steinheim, Germany). 96-well polypropylene microtiter plates were purchased from Greiner Bio-One (Frickenhausen, Germany). Sampling material was obtained from Sarstedt (Nümbrecht, Germany). For sample storage 450 μL CryoTubes™ were purchased from Sarstedt.

### Sample pretreatment

Sample derivatization was performed according to our previously described protocols (Brauer et al. 2011). Shortly, serum samples were diluted 1:10 with methanol for protein precipitation. After centrifugation we placed 10 μL of the supernatant into 96-well polypropylene microtiter plates and diluted it with 100 μL of the internal standard solution. Following evaporation at 70 °C for 40 min, we added 60 μL of 3*n* butanolic-HCl for derivatization at 65 °C for 18 min. The residual solution was again evaporated at 70 °C for 40 min and then reconstituted with 150 μL of the mobile phase (1/1 v/v isopropanol/water). After 15 min of gentle shaking of the microtiter plate at room temperature, we analyzed the samples by flow injection analysis (FIA)-tandem mass spectrometry (MS/MS).

### Tandem mass spectrometry

An API 3000 MS/MS (Applied Biosystems, Germany) equipped with a Turbo Ion Spray Source (TIS) in combination with an HTC Pal autosampler and a PE 200 microgradient pump was used for flow injection analysis (FIA). 25 μL of the sample were directly injected at a flow rate of 80 μL/min in an analysis time of 1.5 min. We detected amino acids by a neutral loss scan of 102 in the mass range of 130–280 or multiple reaction monitoring (MRM). Quantitative analysis using internal standards for 26 amino acids was performed using ChemoView™ 1.4.2 (Applied Biosystems, Germany). A comprehensive overview of mass transitions, internal standards, and performance data for the different amino acids is summarized in Brauer et al. (2011).

### Bioinformatic analysis

**Fig. 1:**
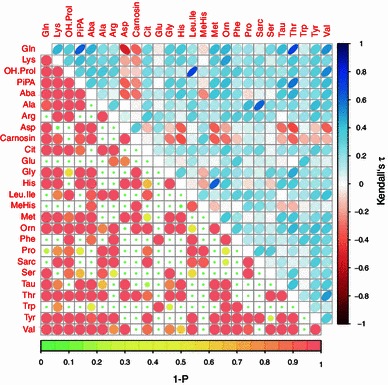
Correlation matrix of the amino acid concentrations (see Murdoch and Chow 1996 for details). In the *upper right part* Kendall’s τ is displayed according to the right-hand legend, in the *lower left part* the corresponding (1 − *P*) values are limned to illustrate the significance of the correlation (cf. legend at the *bottom*). For compound abbreviations, see Table 2

**Fig. 2:**
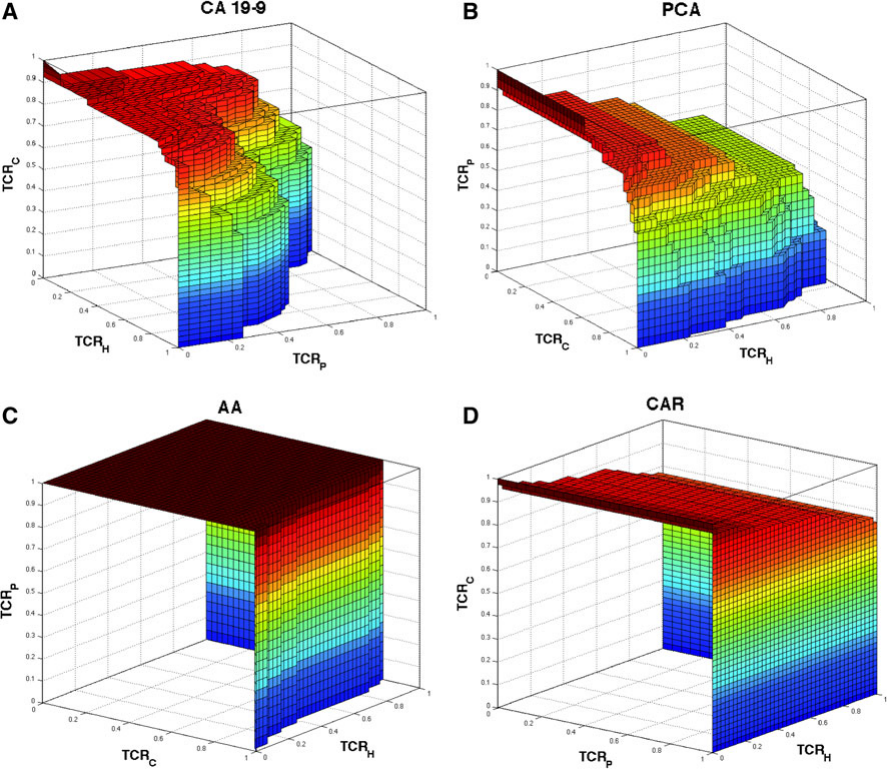
Three-dimensional ROC surfaces depicting true class rates of CA 19-9 alone (**a**), the mlogitBMA predictor model based on PCAs and CA 19-9 (**b**), the mlogitBMA predictor model based on amino acid (AA) concentrations and CA 19-9 (**c**), and the CAR-score based predictor model (**d**, See Section 2) when the three classes/states are assessed simultaneously. The *axes* represent true class rates for healthy controls (H), pancreatitis patients (P), and pancreatic carcinoma patients (C)

**Fig. 3:**
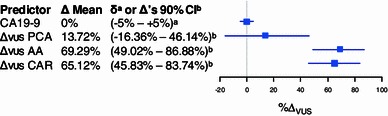
Forest plot of the Δ_VUS_ based on CA 19-9s VUS (Δ_0_, 0.528) as reference with the predefined ±5 % *δ* as *horizontal bars* (*a*). Additionally, the Δ_VUS_ values of the predictors PCA, AA, and CAR are displayed with their (100 − 2 *δ*) % bootstrap CIs (*b*, See Section 2)

Statistical analyses were conducted (unless otherwise stated) using R for Windows (Version 2.14.2) and its related CRAN packages (http://cran.r-project.org/). We tested data for normality by the Anderson–Darling test (nortest) and the gender distribution in the subgroups by the binomial test (stats). The homogeneity of variances of the quantitative routine laboratory data was evaluated with the approximative Fligner–Killeen test (stats), whereas the paired differences were investigated by Games–Howell testing (script source: http://aoki2.si.gunma-u.ac.jp/R/src/tukey.R). Three missing CA 19-9 values were imputed by multiple imputation (MI) with 3 chains of imputation (which were averaged thereafter), a $\hat{\text{R}}$ value of 1.1, and bootstrap as random imputation method until conversion (after 7111 iterations). Three pancreatitis samples with non-random missing data as a consequence of insufficient sample volume were excluded from further analysis. The selectivity of single amino acid concentrations was assessed in all disease states simultaneously via the volume under ROC surface (VUS), which is the three-dimensional analogue of AUROC analysis (Nakas and Yiannoutsos 2004). The VUS’ and their associated confidence intervals were calculated nonparametrically using $B = 2 ,000$ bootstraps and 50 k subdivisions on the amino acid concentrations (DiagTest3Grp). As we assumed a high degree of collinearity in the amino acid concentrations, we computed Kendall’s τ as well as its Hochberg-adjusted significance (ltm, corrplot) and plotted the correlation matrix (Fig. 1). Based on our previous results indicating that marker models comprising combinations of different amino acids and/or CA 19-9 might be superior to single amino acids and/or CA 19-9 with respect to their selectivity (Leichtle et al. 2012), we also evaluated combined models. These models consisted on the one hand of the conventional tumor marker CA 19-9 combined with different principal components (PC1, PC2, …) of the different amino acid concentrations to control for multicollinearity, which is a significant constraint on variable selection (Leigh 1988). On the other hand we also combined CA 19-9 with mere amino acid concentrations to avoid potentially unnecessary transformation steps prior to modeling. After Yeo–Johnson transformation (car) of the amino acid concentrations, we generated PCs (princomp and factoMineR), from which the first six PCs had eigenvalues >1.0 and cumulatively covered 78.7 % of the variance. For the modeling in a three-state design, we merged sample set A with C and obtained one tumor group, one control group and one pancreatitis group (set D). In the second step we used CA 19-9 alone and combined with the PCs of the amino acid concentrations as well as with mere concentrations for Bayes-averaged multinomial logit modeling [mlogitBMA, bic.mlogit(mlogitBMA)] using Begg and Gray approximation. We validated the latter model by CAR [‘Correlation-Adjusted (marginal) coRrelation’] scores (care) assuming empirical values of 1.0, 0.3, and 0.1 as responses of the pancreatic carcinoma, the healthy control, and the pancreatitis groups, respectively, in a CAR model truncated to a number of variables comparable to the penalized multinomial logit model. We computed the VUS for the four predictors, namely CA 19-9, the PCA-based mlogitBMA-predictor (PCA), the amino acid concentration-based mlogitBMA-predictor (AA), and the amino acid concentration-based CAR-model-predictor (CAR) analogously to the VUS values of the amino acid concentrations. The ROC surface plots (Fig. 2) were drawn using MATLAB (The MathWorks, Natick, MA, USA). Since significant differences of the VUS between predictors and CA 19-9 do not imply inferiority or superiority, we performed non-inferiority and superiority testing applying bootstrap techniques on Δ_VUS_ ($B_{\text{outer}} = 1 ,000$, boot, $B_{\text{inner}} = 1 ,000$, DiagTest3Grp, UBELIX Cluster of the University of Bern). We constructed the corresponding CIs and tested for the overlap of Δ_0_ and the Δ_VUS_’ CI according to the methods proposed by Liu et al. (2006), Tunes da Silva et al. (2009), and Lesaffre (2008) at a predefined *δ* level of 5 % which was considered to be medically reasonable designing this and a previous study (Leichtle et al. 2012). We visualized the performance data in a forest plot [Fig. 3, forestplot(rmeta), R version 2.15.0, cf. Mascha (2010)].

### Ethics

The study was approved by the Ethics Committee of the Medical Faculty of the University of Leipzig (Reg. No. 013-2005) and it fulfills the requirements of the Helsinki declaration. All study subjects gave written informed consent to participate in the study.

## Results and discussion

### Descriptives

We collected serum samples of 40 (20 males/20 females) pancreatic carcinoma patients, 26 (22 males/4 females, $P_{\text{binomial}} = 0.000 5$) pancreatitis patients, and 40 (20 males/20 females) healthy controls. Table 1 displays the distributions of age, BMI, UICC cancer staging of the patients, the CA 19-9, bilirubin, and HbA1c concentrations in the three different sample sets. Of 26 amino acid concentrations, we found only four (arginine, glutamic acid, phenylalanine, and tryptophan) unaltered between the study groups (Table 2). Several amino acid concentrations were non-normally distributed. In order to detect sample set-specific alterations in the values, we also compared the amino acid concentrations of the tumor patients and the healthy control group between the two sample sets and found no significant differences (Table 2).

**Table 2:** Inter-group significance (*P* values) of the differences in CA 19-9 and amino acid concentrations as evaluated by Games–Howell testing and the homogeneity of variances as determined by Fligner–Killeen testing (column *P*)

Compound	Ap:Ac	Ap:Cp^a^	Ap:Cc	Ap:D	Ac:Cp	Ac:Cc^a^	Ac:D	Cp:Cc	Cp:D	Cc:D	*P*
CA19-9^b^	**0.003**	0.692	**0.003**	**0.006**	**0.030**	0.753	0.058	**0.032**	0.069	0.107	**0.000**
Glutamine (Gln); (CID 5961)	**0.000**	0.239	**0.000**	**0.000**	**0.007**	0.962	**0.000**	**0.012**	**0.000**	**0.000**	**0.000**
Lysine (Lys) (CID 5962)^b^	0.791	0.876	0.971	**0.029**	0.319	0.972	**0.005**	0.523	0.259	**0.004**	0.865
Hydroxyproline (OH.Prol) (CID 5810)	0.050	0.932	0.102	0.996	0.271	1.000	**0.015**	0.371	0.755	**0.043**	0.706
Pipecolic acid (PiPA) (CID 849)	**0.001**	0.245	**0.000**	**0.020**	0.605	0.982	**0.000**	0.360	**0.002**	**0.000**	0.371
Abscisic acid (Aba) (CID 5280896)	0.440	0.999	0.545	**0.000**	0.530	0.999	**0.000**	0.646	**0.000**	**0.000**	**0.000**
Alanine (Ala) (CID 5950)	0.067	1.000	0.383	0.998	**0.019**	0.917	0.118	0.212	0.989	0.547	0.447
*Arginine (Arg)* (CID 6322)	0.999	0.998	0.950	0.932	1.000	0.991	0.980	0.994	0.984	1.000	0.759
Aspartic acid (Asp) (CID 5960)	0.121	0.982	0.120	**0.000**	0.138	1.000	**0.000**	0.136	**0.000**	**0.000**	**0.000**
Carnosin (CID 439224)^b^	0.411	0.377	0.216	**0.000**	1.000	1.000	**0.000**	0.997	**0.000**	**0.000**	**0.000**
Citrulline (Cit) (CID 9750)	0.095	0.994	0.679	1.000	0.160	0.330	**0.037**	0.884	0.989	0.523	0.481
*Glutamic acid (Glu)* (CID 33032)	0.881	0.266	0.757	0.602	0.101	0.998	0.224	0.075	0.897	0.168	**0.000**
Glycine (Gly) (CID 750)	0.890	1.000	0.984	0.113	0.831	0.993	**0.011**	0.966	0.079	**0.019**	0.879
Histidine (His) (CID 6274)^b^	0.995	1.000	0.999	0.068	0.987	0.971	0.087	1.000	**0.009**	0.057	**0.000**
Leucine/Isoleucine (Leu.Ile) (CID 6106/CID 6306)	**0.033**	0.999	**0.040**	0.134	**0.046**	1.000	0.971	0.056	0.183	0.954	0.883
Methylhistidine (MeHis) (CID 64969)	**0.049**	0.907	0.289	**0.000**	0.623	0.877	**0.000**	0.956	**0.000**	**0.000**	**0.001**
Methionine (Met) (CID 6137)^b^	1.000	0.999	1.000	0.604	0.978	1.000	0.188	0.996	**0.021**	0.314	0.050
Ornithine (Orn) (CID 6262)	0.199	0.531	0.708	**0.015**	0.953	0.846	**0.000**	0.998	**0.001**	**0.002**	0.722
*Phenylalanine (Phe)* (CID 994)^b^	0.962	0.995	0.967	1.000	1.000	1.000	0.975	1.000	0.993	0.972	0.069
Proline (Pro) (CID 8988)	0.051	0.957	**0.049**	**0.022**	0.163	1.000	0.971	0.153	0.187	0.957	**0.009**
Sarcosine (Sarc) (CID 1088)^b^	0.272	0.507	0.953	0.786	**0.002**	0.681	0.999	0.119	0.173	0.967	0.212
Serine (Ser) (CID 5951)	0.091	0.380	**0.011**	**0.018**	0.931	0.816	0.998	0.382	0.746	0.891	0.446
Taurine (Tau) (CID 1123)	**0.003**	0.053	**0.016**	**0.001**	0.595	1.000	**0.000**	0.784	**0.000**	**0.000**	0.091
Threonine (Thr) (CID 6288)	**0.008**	0.886	0.051	**0.000**	0.086	1.000	**0.000**	0.246	**0.000**	**0.000**	**0.000**
*Tryptophan (Trp)* (CID 6305)^b^	0.589	0.705	0.151	0.058	0.999	1.000	0.945	0.985	0.798	0.931	0.189
Tyrosine (Tyr) (CID 6057)	**0.041**	0.818	0.056	1.000	0.421	1.000	0.050	0.510	0.840	0.068	0.855
Valine (Val) (CID 6287)	**0.000**	0.331	**0.016**	0.475	0.116	0.999	**0.000**	0.316	**0.029**	**0.002**	0.084

Sets A, C, and D (only pancreatitis) with pancreatic carcinoma patients (p) and controls (c). *P* values <0.05 are shown as *bold numbers*. Amino acids with no significant difference in any group comparison are displayed in *italics*. Metabolites are identified by their PubChem Compound ID (CID)

^a^Comparisons of subjects of the same class in different sets

^b^Signifies deviation from normal distribution in at least one subgroup as evaluated by Anderson–Darling testing

### Correlations

We evaluated the multicollinearity of the amino acid concentrations by generating their correlation matrix (Kendall’s τ and its Hochberg-adjusted significance). Kendall’s τ values ranged between −0.516 (aspartic acid with glutamine) and 0.709 (threonine with glutamine), the *P* values between 0.001 and 0.97 (Fig. 1).

### Modeling

We hypothesized that combinatory markers including several amino acids and/or CA19-9 might have additive or even multiplicative effects and thus be superior to single amino acids and/or CA 19-9 in diagnostics of pancreatic cancer (Brand et al. 2011). Therefore, in addition to evaluating the single VUS of the amino acid concentrations, we also generated models based on CA 19-9 combined with PCs and Bayesian multinomial logit model averaging (mlogitBMA) as well as on CA 19-9 conjoined with amino acid concentrations. Furthermore, we used models based on CAR scores to evaluate their three-class selectivity in comparison with that of single amino acid concentrations and CA 19-9 as a validation method for the mlogitBMA models. The best PC-based mlogitBMA-model comprised CA 19-9 and PC2. The best amino acid concentration-based mlogitBMA-model contained CA 19-9 and aspartic acid, which both were also contained in the truncated amino acid concentration-based CAR score-model. To evaluate the selectivity of both, amino acid concentrations and modeled predictors, we computed their volume under the ROC surface (VUS) (Table 3). The VUS of the amino acid concentrations spanned from 0.180 (arginine, 95 % CI 0.106–0.270) to 0.850 (glutamine, 95 % CI 0.761–0.929). The VUS of CA19-9 was 0.528 (95 % CI 0.400–0.654), and the predictors PCA, AA, and CAR had VUSs of 0.604 (95 % CI 0.446–0.745), 0.891 (95 % CI 0.794–0.968), and 0.871 (95 % CI 0.776–0.952), respectively. For a random classifier, the VUS could be geometrically determined (Landgrebe and Duin 2007) with a value of $1.\bar{6}.$ To illustrate the selectivity of CA 19-9 and the predictors, we generated true class rate-plots depicting the ROC surface (Fig. 2).

**Table 3:** Volume under receiver operator characteristics curve (VUS) and 95 % confidence intervals of the amino acid and CA 19-9 concentrations as well as of a random classifier [cf. Landgrebe and Duin (2007)] with respect to the discriminatory power between pancreatic cancer patients, healthy controls, and pancreatitis patients

Compound	VUS	Low 95 %CI	High 95 %CI
Gln	0.8500	0.7606	0.9288
Thr	0.7531	0.6431	0.8581
Asp	0.7156	0.6006	0.8225
PiPA	0.6961	0.5934	0.7976
Aba	0.6305	0.5097	0.7461
Tau	0.6128	0.4869	0.7296
MeHis	0.5946	0.4647	0.7224
His	0.5636	0.4328	0.6915
Carnosin	0.5509	0.4233	0.6695
CA19-9	0.5282	0.3996	0.6536
Val	0.5246	0.405	0.6493
Met	0.4966	0.3685	0.6231
Orn	0.4663	0.3528	0.5928
Lys	0.4375	0.3299	0.5561
Gly	0.4096	0.2969	0.5313
Tyr	0.3457	0.2353	0.4749
OH-Prol	0.3397	0.233	0.4562
Ser	0.3239	0.2228	0.4349
Leu/Ile	0.3157	0.217	0.4305
Cit	0.2845	0.1789	0.4022
Trp	0.2791	0.1789	0.3922
Pro	0.2758	0.1705	0.3957
Sarc	0.2607	0.1547	0.3822
Ala	0.2591	0.1677	0.3607
Phe	0.232	0.145	0.3395
Glu	0.2273	0.1419	0.3164
Arg	0.1801	0.1063	0.2698
Random	$1.\bar{6}$ (${1 \mathord{\left/ {\vphantom {1 6}} \right. \kern-0pt} 6}$)		

For compound abbreviations, see Table 2

### Non-inferiority and superiority testing

Since it is generally accepted that significant difference alone might not be an adequate measure of non-inferiority or superiority, we sequentially tested for both with an a priori-defined acceptance criterion (equivalence limit) of $\delta = 5\,\%$ Δ_VUS_. We computed the lower and upper limits of the (100 − 2*δ*) % bootstrap confidence interval of the estimated Δ_VUS_ as proposed by Liu et al. (2006). The results are shown in Fig. 3. Following the criteria by Mascha (2010) we deduced non-inferiority for predictors AA and CAR compared to CA 19-9. Furthermore, superiority of the AA and CAR predictors over CA 19-9 alone was derived in a second step, since the lower CI of Δ_VUS_ was positive.

“The ideal biological marker(s) for cancer risk assessment and early detection must have high sensitivity and specificity, be found in a biosample obtained using minimally invasive procedures, and be analyzed using a highthroughput, cost-effective assay.” These requirements stated by Van and Veenstra (2009) are challenging to fulfill. Particularly, in the case of pancreatic cancer diagnostics this challenge is even bigger due to the number of differential diagnoses, which are difficult to discern from malignant disease even for experienced clinicians (Gong et al. 2012). Furthermore, chronic pancreatitis patients also have a 15-fold higher risk than the general population to develop pancreatic cancer (Huggett and Pereira 2011). In order to identify biomarkers capable of discriminating different disease states, we designed a study including pancreatic cancer patients and healthy controls of two independently collected sample sets as well as an additional group of pancreatitis patients, since the principal feasibility of the metabolomic approach to pancreatic cancer was recently shown (Bathe et al. 2011; Tesiram et al. 2012; Zhang et al. 2012). Samples were processed following highly standardized preanalytical protocols and applying a routinely used tandem mass spectrometric technique. By comparing the sample groups, we found 22 of 26 amino acids altered in at least one out of ten possible comparisons. The number of different metabolites is comparable to that given by Bathe et al. (2011) who found 22 of 58 metabolites significantly different between malignant and benign pancreatic disease applying ^1^H NMR and 2D NMR spectroscopy, with OuYang et al.’s (2011) ^1^H NMR spectroscopy results showing significant alterations of at least 8 metabolites between only 17 pancreatic carcinoma patients and 25 healthy controls. It is consistent with Urajama et al.’s (2010) combined GC/TOF-MS, LC/ESI-MS, and LC/LTQ-Orbitrap study revealing 26 significantly different metabolites in a comparison of 5 pancreatic cancer samples and 5 mixed pancreatitis/healthy controls, and with Nishiumi et al.’s (2010) GC–MS investigations based on 21 pancreatic cancer patients and 9 healthy volunteers identifying 18 of 60 metabolites as significantly different. In addition to the inter-class comparisons of the different sample sets, we also evaluated the inter-sample set differences in the respective classes (cancer_A_ − cancer_C_ and control_A_ − control_C_) and found no significant differences. Since the sample groups were homogeneous, we preferred a joint analysis in the modeling approach over a split-half design to keep the degree of random error as low as possible (Knottnerus and Muris 2003; Ransohoff and Gourlay 2010). Although the previously published metabolome profiling studies of pancreatic carcinoma are heterogeneous regarding the used mass-spectrometric techniques and the studied metabolites, they all share canonical variance-based evaluation strategies with two-class comparisons. Additionally, only one of the studies (Bathe et al. 2011) assessed the selectivity (e.g. AUROC or VUS analyses) of the marker metabolites. Our aim was to perform a comprehensive data analysis that also allows a clear interpretation of the diagnostic value of the markers (Leichtle et al. 2012). To this end, we implemented four unexampled features in our bioinformatic pipeline: (1) The computation of three-class VUS values of the single amino acid concentrations as an integral selectivity measure, (2) a Bayesian multinomial logit model averaging procedure to extend the previously used binomial logistic regression modeling (Leichtle et al. 2012) on the three-class study design to generate multi-marker panels (including CA 19-9), (3) the VUS-based analysis of the panel predictors, and, finally, (4) their non-inferiority and superiority determination. The VUS values of the single amino acid concentrations ranged from 0.18 slightly above a random classifier to 0.85 (glutamine), which is close to the best panel predictors. As none of the previous metabolite profiling studies on pancreatic cancer performed VUS analysis, we can only rely on the utterly inconsistent *P* values they present, in Urayama et al.’s (2010) case 0.000021, or in Nishiumi et al.’s (2010) 0.97 for glutamine. CA 19-9 alone reached the 10th rank, which is probably attributable to its low selectivity between benign and malign pancreatic diseases (Fig. 2a). Since our previous investigations (Leichtle et al. 2012) indicated a high degree of multicollinearity in the amino acid concentrations, which is known to impede many feature selection techniques (Jesneck et al. 2009; Leigh 1988), we set up a Kendall’s correlation matrix to visualize the multicollinearity and its significance. As expected, the full range of correlation spanned from −0.516 to 0.709, which supported the inclusion of the frequently recommended PC-based analysis approach, albeit it has been shown that variance-based techniques might not always yield optimal predictors (Leichtle et al. 2012). To compute robust predictive multi-metabolite marker panels, we combined CA 19-9 and the PCs as well as CA 19-9 and the mere amino acid concentrations and used a Bayesian multinomial logit model averaging procedure for our categorical three-class study design (Robin et al. 2009). The first model consisted of CA 19-9 and PC2 providing a two-marker “panel” predictor (PCA) with a VUS of 0.604. The omission of PC1 and preference of PC2 with less contribution to explained variance during the mlogitBMA procedure is an astounding finding possibly reflecting a predilection of variables sharing covariance with CA 19-9. The second model based on amino acid concentrations included CA 19-9 and aspartic acid providing a two-marker “panel” predictor (AA) with a VUS of 0.891. Our results indicate that CA 19-9 provides the selectivity mainly for the discrimination between healthy controls and pancreatic cancer patients (Table 2), whilst aspartic acid predominantly contributed to the identification of pancreatitis patients. Nishiumi et al. (2010) reported a borderline significant *P* value of 0.075 for aspartic acid, whereas Urayama et al. (2010), OuYang et al. (2011) and Bathe et al. (2011) did not mention significant differences. Our results and panel predictors, however, require extremely cautious interpretation since in a previous study an analytical variability >25 % was observed for aspartic acid (Brauer et al. 2011). On the other hand, regarding the substantial impact of especially pancreatic disease on nutrition, it was not unexpected to find models different to those of our previous study on colorectal cancer (Leichtle et al. 2012). The mechanisms disturbing amino acid homeostasis and enabling the discrimination of pancreatic cancer patients from pancreatitis patients on the basis of metabolite profiles are not entirely elucidated. Schrader et al. (2009) suggested—apart from malnutrition—mainly inflammatory effects and pointed at the inverse relationship between the circulating amino acid concentrations and the degree of inflammation present e.g. in hemodialysis patients. Whether increased tumor-associated proteolytic activity (Findeisen and Neumaier 2012) contributes not only to the generation of specific peptide decay profiles, but also to the specificity of the amino acid profiles is still unknown. To validate our results and the Bayesian modeling approach, we also applied model selection techniques based on CAR scores (Zuber and Strimmer 2011) as a non-Bayesian linear alternative. Since our study covered three—more or less—independent classes, we could neither rely on a binary (CAT score) nor on a metric (CAR score) response. Therefore we assumed empirical values of 1.0, 0.3, and 0.1 as “responses” of the respective groups while acknowledging that such a procedure might be somewhat artificial and not necessarily justified by the underlying pathophysiology. To gain a comparable number of model variables as in the penalized mlogitBMA-model and thereby an at least limited comparability, we used a two-predictor CAR model including CA 19-9 and aspartic acid. The CAR panel predictor had a VUS of 0.871 similar to the value obtained with the Bayesian modeling approach. As the final evaluation step, we performed a two-step non-inferiority and superiority testing based on the bootstrapped Δ_VUS_ and on a ± *δ* equivalence range of 5 % as outlined in a previous study (Leichtle et al. 2012). CA 19-9’s VUS ± *δ* served as reference we tested the other predictors’ Δ_VUS_ against. In the first step, we observed non-inferiority only for the AA and CAR panel predictors, but not for the PCA panel predictor, whereas in the second step, we determined superiority of AA and CAR panel predictors (Fig. 3). These encouraging results indicate an improved selectivity of the models compared to CA 19-9 alone. Our study has several limitations to be considered. First, we merged the sample sets A and C to keep the degree of random error as low as possible in our modeling analysis (Knottnerus and Muris 2003). However, the “external” validity of the results could not thus be evaluated (Ransohoff and Gourlay 2010). Therefore, subsequent studies are necessary in order to assess the generalizability of our predictor models. Second, due to high preanalytical standardization and refinement of our bioinformatic methodology, the variability of the analytical method itself might have become the main source of bias. With our study design and evaluation strategy, we probably have reached an analytical boundary, that still requires substantial improvements (Hori and Gambhir 2011). Therefore, new analytical techniques are necessary to reach both, superior sensitivity and stability at the same time. The third limitation of the study originates from the strong penalization of our Bayesian modeling approach: The predictor panels generated by the mlogitBMA procedure were both two-component panels consisting of CA 19-9 and another variable. Especially in the case of PC-based modeling and the selection of the second PC while leaving out the first, a considerable amount of selectivity might have been lost. On the other hand, the amino acid concentration-based model was superior in selectivity [without taking misclassification costs into account (Klawonn et al. 2011)], suggesting that PCs might not serve as optimal modeling variables when Occam’s razor is strictly availed. Finally, rather than proposing a superior diagnostic metabolite model or “meta-marker” our results suggest that our bioinformatic framework combined with a methodology refined to sufficient sensitivity and stability might provide a valuable diagnostic tool for metabolic profiling even in the three-class differentiation dilemma of health, inflammation, and malignancy.

## Short summary

Multi-marker panels have been suggested to improve the selectivity of pancreatic cancer diagnostics and its differentiation from various benign lesions. However, a comprehensive framework for the statistical evaluation of marker panels in a multi-class setting has not yet been established.

Using a disease model encompassing pancreatic cancer, pancreatitis, and healthy controls, 106 standardized serum samples, and metabolic profiling, we generated models to discriminate between the three study groups.

Multi-marker models are superior to the conventional tumor marker CA 19-9 in simultaneously differentiating between pancreatic cancer, pancreatitis, and healthy controls.

Our comprehensive bioinformatic approach provides a novel framework to address a common diagnostic challenge, and thus paves the way for biomarker validation in a clinical three-class setting.

## Abbreviations

^1^H NMR: Proton nuclear magnetic resonance (spectrometry)
2D NMR: 2-Dimensional nuclear magnetic resonance (spectrometry)
AU(RO)C: Area under the (receiver operator characteristics) curve
mlogitBMA: Bayesian multinomial logit model averaging
CA 19-9: Carbohydrate antigen 19-9
CAR scores: ‘Correlation-adjusted (marginal) correlation’ scores
CEA: Carcinoembryonic antigen
GC–MS: Gas chromatography mass spectrometry
GC/TOF–MS: Gas chromatography/time of flight mass spectrometry
ICAM-1: Intracellular adhesion molecule 1
LC/ESI-MS: Liquid chromatography/electrospray ionization mass spectrometry
LC/LTQ-Orbitrap: Liquid chromatography/linear ion trap combined with an orbitrap (mass spectrometry)
OPG: Osteoprotegerin
PC(A): Principal component (analysis)
TIMP-1: TIMP metallopeptidase inhibitor 1
VUS: Volume under (ROC) surface
